# 1810. Noma (Cancrum oris): World Health Organisation's latest neglected tropical disease?

**DOI:** 10.1093/ofid/ofad500.1639

**Published:** 2023-11-27

**Authors:** Ramat O Braimah, Azeez Butali, Peter Mossey, Thompson Wendy, Abdurrazaq Taiwo, Modupe Coker, Mujtaba Bala, Adebayo Ibikunle, Shafiu Abdullah, Abubakar Bello

**Affiliations:** Faculty of Dental Sciences,, Sokoto, Sokoto, Nigeria; Iowa Institute of Oral Health Research and Iowa Institute of Human Genetics, University of Iowa, Iowa, Iowa; Dundee University Dental School, Dundee, Scotland, United Kingdom; Cumbria And North East Commissioning Hub, Cumbria, England, United Kingdom; Faculty of Dental Sciences, Usmanu Danfodiyo University, Sokoto, Sokoto, Sokoto, Nigeria; Rutgers State University of New Jersey, New Jersey, New Jersey; Usmanu Danfodiyo University Teaching Hospital, Sokoto, Sokoto, Nigeria; Usmanu Danfodiyo University Teaching Hospital, Sokoto, Sokoto, Nigeria; Noma Children Hospital, Sokoto, Sokoto, Nigeria; Noma Children Hospital, Sokoto, sokoto, Sokoto, Nigeria

## Abstract

**Background:**

Noma, also known as cancrum oris, necrotizing ulcerative stomatitis or gangrenous stomatitis, is a rapidly progressive and often fatal infection of the mouth and face. This disease predominantly affects children between the ages of 2 and 6 years old in poorly developed countries around the world where adequate nutrition, sanitation and cleanliness are lacking. Northwest Nigeria has a particularly high prevalence of the Noma.

Noma starts as gum disease and, without effective treatment (including antibiotics), around 90% die within two weeks and as such noma eminently fits the definition of a neglected tropical disease (NTD). The World Health Organization (WHO) has described 5-stages of the disease to include: Stage 1 (Acute Necrotizing Ulcerative Gingivitis), Stage 2 (Edematous), Stage 3 (Gangrenous), Stage 4 (Scarring) and Stage 5 (Late sequelae).

Stage 1 noma - Acute necrotizing ulcerative gingivitis (necrotizing periodontal disease)

**Figure:**
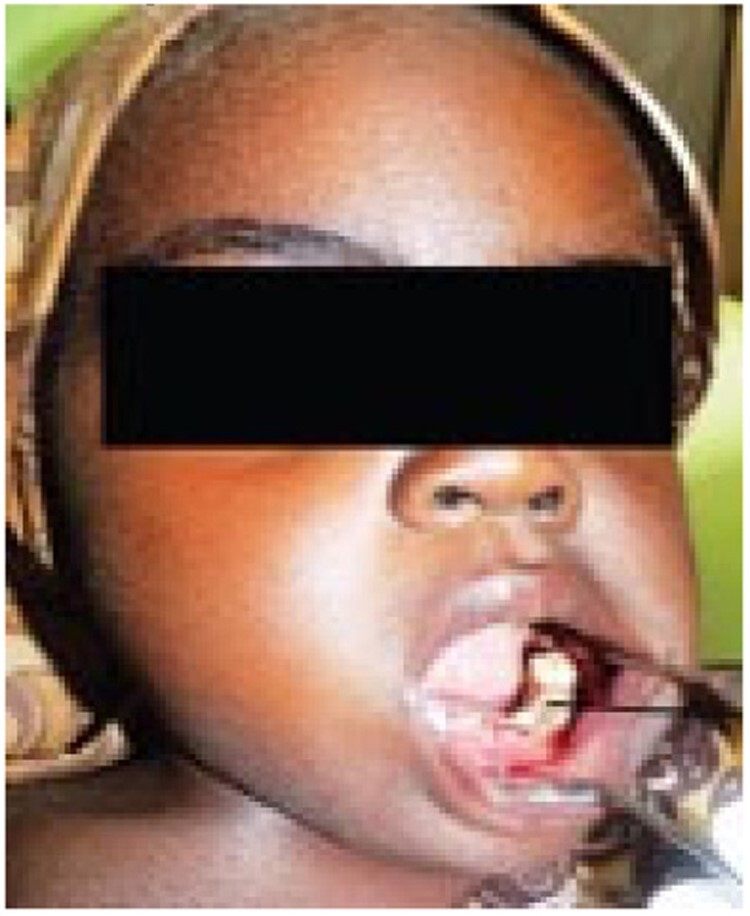

Stage 1 noma - Acute necrotizing ulcerative gingivitis (necrotizing periodontal disease)

**Figure:**
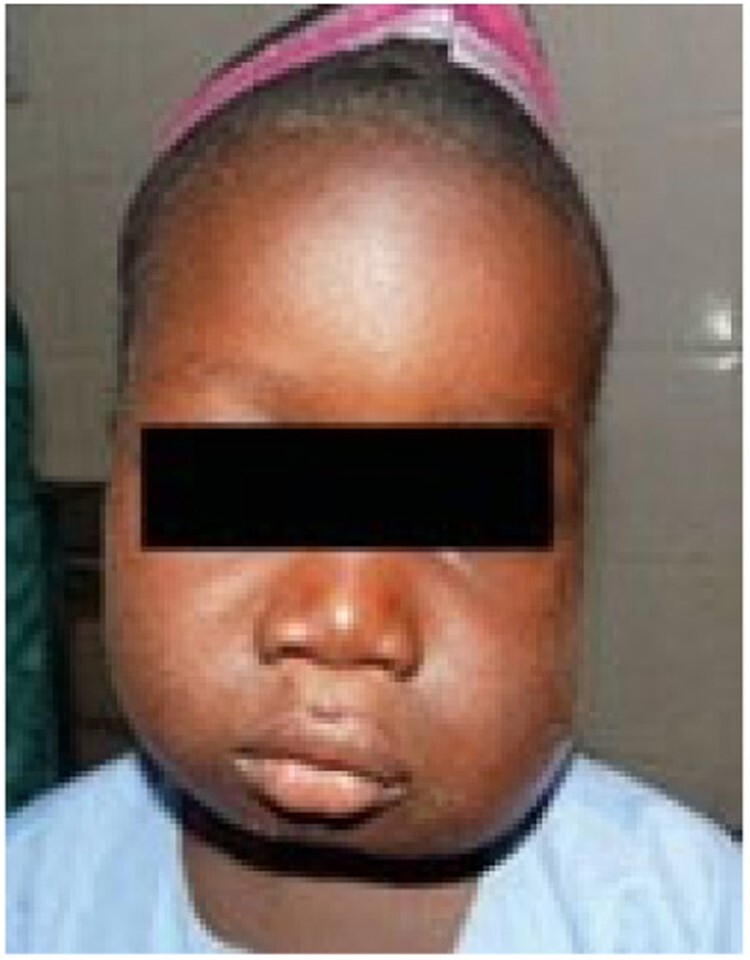

Stage 2 noma – Edema

**Methods:**

This study describes the patients who presented at hospitals in Sokoto, Nigeria between 2014 and 2022. Data were collected at Usmanu Danfodiyo University Teaching Hospital, Noma Children’s Hospital and State Specialist Hospital. The data are supplemented with exemplar images of cases which presented in each stage of noma.

Stage 3 noma - Gangrenous

**Figure:**
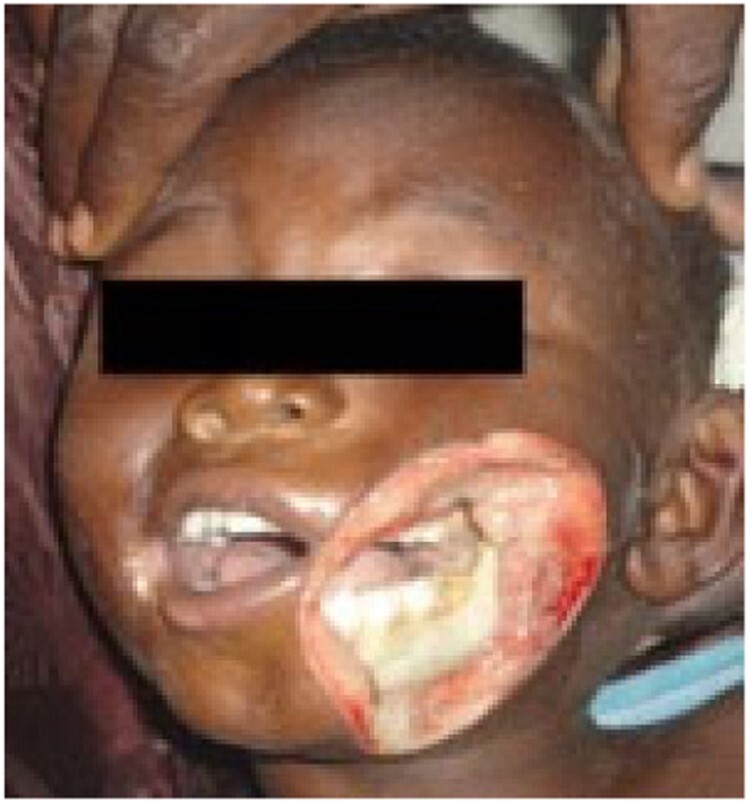

Stage 4 noma – Scarring

**Figure:**
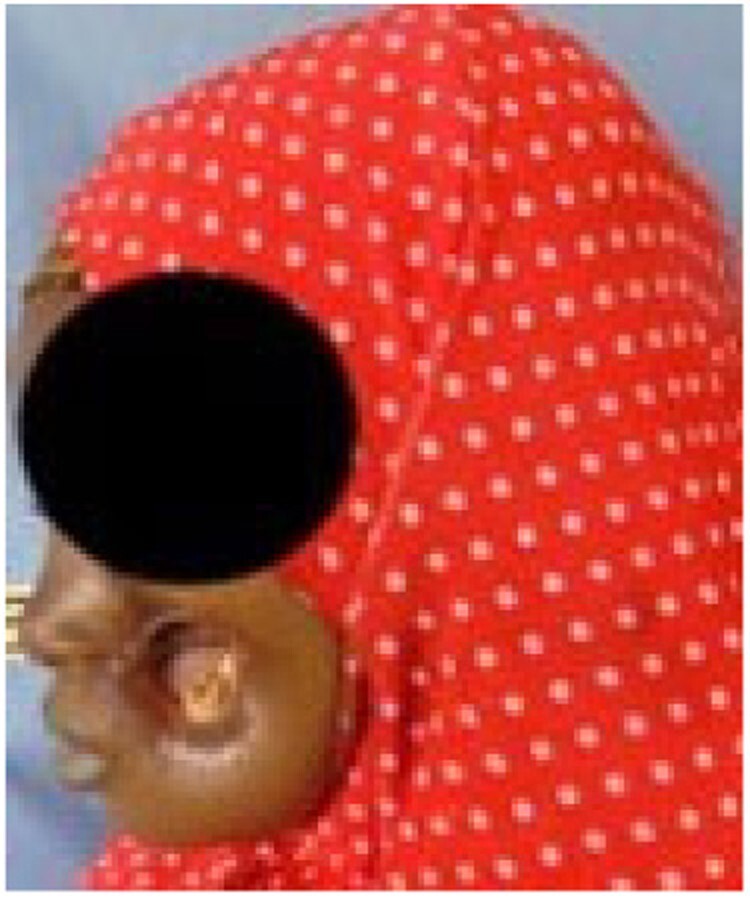

**Results:**

Since 2014, a total of 1,147 patients have been admitted. 165 (14.4%) patients presented with Stage 1 noma and were enrolled in the Ambulatory Therapeutic Feeding Centre (ATFC) (Figure 1). 125 (10.9%) patients presented with Stage 2 or 3 noma(see Figure 2)and were admitted to Inpatient Therapeutic Feeding Centres (ITFC). 845 (73.7%) patients presented with Stage 4 or 5 noma (see Figure 3) had various surgical interventions.

One case of twins both presenting with noma was seen. One presented with Stage 3 noma and the other was in Stage 4 . Just 11 (1.0%) cases were adults, all of whom presented with Stage 3 noma.

Stage 5 noma – late sequelae

**Figure:**
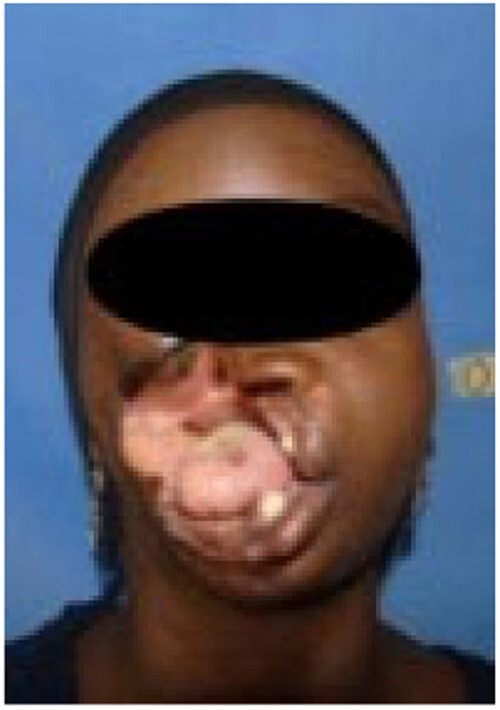

**Conclusion:**

Noma represents a major health inequality and has a relatively high prevalence in Nigeria. While previous studies have identified possible risk factors, there is a paucity of knowledge about the etio-pathogenesis, prevention and treatment of each phase. The designation of noma as a neglected tropical disease would focus attention on the condition and would release funding for further research to address these knowledge gaps.

**Disclosures:**

**All Authors**: No reported disclosures

